# Molecular and Serological Detection of Vector-Borne Pathogens Responsible for Equine Piroplasmosis in Europe between 2008 and 2021

**DOI:** 10.3390/microorganisms12040816

**Published:** 2024-04-17

**Authors:** Carla Wiebke Axt, Andrea Springer, Christina Strube, Clarissa Jung, Torsten J. Naucke, Elisabeth Müller, Ingo Schäfer

**Affiliations:** 1 LABOKLIN GmbH and Co. KG, Steubenstraße 4, 97688 Bad Kissingen, Germany; axt@laboklin.com (C.W.A.); mueller@laboklin.com (E.M.); 2 Institute for Parasitology, Centre for Infection Medicine, University of Veterinary Medicine Hannover, 30559 Hannover, Germany

**Keywords:** tick-borne disease, *Babesia caballi*, *Theileria equi*, epidemiology, horses

## Abstract

Equine piroplasmosis (EP) is caused by *Theileria* (*T*.) *equi* and/or *Babesia* (*B*.) *caballi*. The aim was to assess the percentage of positive test results for EP in horses in Europe and to identify risk factors for pathogen contact/infection. This study included results from PCR and competitive enzyme-linked immunosorbent assay testing requested by European veterinarians between 2008 and 2021. Binary bivariate logistic regression was used to analyze risk factors. A total of 4060 horses were included. PCR testing was positive in 9.7% (154/1589), serology for *T. equi* in 15.2% (393/2591) and for *B. caballi* in 6.8% (175/2578). The odds of positive serology increased by 6.8% (*B. caballi*, *p* = 0.008) and 9.5% (*T. equi*, *p* < 0.001) each year. Regionality had a statistically significant impact on PCR (Eastern *p* = 0.047/OR = 1.605; Southern *p* = 0.029/OR = 1.451; Central *p* = 0.007/OR = 0.617) and serological testing for *T. equi* (Southern *p* < 0.001/OR = 2.521; Central *p* < 0.001/OR = 0.537; Northern *p* = 0.003/OR = 0.462), as well as breeds on seroprevalence of *B. caballi* (heavy horses: *p* = 0.016/OR = 2.239) and *T. equi* (ponies: *p* = 0.007/OR = 0.340; warmbloods: *p* = 0.025/OR = 1.602). In conclusion, there was a significant geographical impact on the results of PCR and serology, consistent with known vector habitats. The rising numbers of horses tested serologically positive highlights the importance of surveillance.

## 1. Introduction

Equine piroplasmosis (EP) is a tick-borne disease seen in horses, donkeys, mules, and zebras, caused by the protozoan parasites *Babesia* (*B*.) *caballi* and *Theileria* (*T*.) *equi* (formerly *B. equi*) [1]. Both pathogens are mainly transmitted by hard ticks like *Hyalomma* spp., *Rhipicephalus* spp., and *Dermacentor* spp. [2]. Transplacental infection with *T. equi* can occur in carrier mares, causing abortion or neonatal piroplasmosis in the foal [3,4,5,6,7,8]. There have also been reports of other possible transmission routes, including iatrogenic transmission by contaminated needles and syringes, blood transfusion, and surgical instruments [5]. Horses may be latently infected with *T. equi* for years during which they serve as reservoirs [9]. *Theileria equi* is transmitted transstadially in tick populations [10]. Ticks are considered reservoir hosts for *B. caballi*, with transstadial and transovarial transmission [11,12].

EP is endemic in tropical and subtropical regions in Asia, South and Central America, parts of the Southern USA, Africa, and Southern Europe [13]. The frequent movement of horses worldwide has created a risk of pathogen introduction in previously non-endemic countries like Canada, New Zealand, Australia, and Singapore, as well as other US states [14,15]. In Europe, positive PCR results for *B. caballi*/*T. equi* have been reported in horses in the Netherlands (0.0%/1.6%) [16], Poland (-/7.2%) [17], Hungary (0.0%/15.1%) [18], Romania (2.2%/20.3%) [19], the central Balkans (2.1%/22.5%) [20], Croatia (3.6%/13.2%) [21], and Italy (10.3%/70.3%) [22]. There have also been reports of seropositive horses in central Germany (0.3%/6.1%) [23], Switzerland (2.9%/5.8%) [24], the Netherlands (0.2%/0.3%) [25], the Czech Republic (0.4%/1.1%) [26], the United Kingdom (4.4%/5.9%) [27], Ireland (1.5%/2.5%) [28], Greece (1.1%/9.2%) [29], Italy (8.9%/39.8) [22], Spain (6.5%/53.7%) [30], Romania (-/12.8%) [31], and France (13.2%/9.5%) [32]. Furthermore, there have been rare reports of autochthonous infections with *T. equi* in two horses in the Netherlands [16], one horse in Austria [33], and in Germany for one [34] as well as three horses [33].

The primary diagnostic method for EP is the microscopy of blood smears with the identification of parasites in erythrocytes during the acute stage of infection. However, false negative results are common in the blood smear analysis as the level of parasitemia often remains low even in acute infections [35]. Molecular testing using PCR is more sensitive especially in acute stages of infection due to a detection limit of 10^−7^% parasitized erythrocytes. Additionally, PCR testing is able to clearly differentiate between species and genotypes [7]. In vitro cultures of equine piroplasms from peripheral blood are possible but of limited value due to the relatively long turnover of results and the requirement for fresh blood samples and skilled personnel [2,14,15,35,36,37,38,39,40,41]. While less suited for the diagnosis of acute illness, serological assays are crucial for the detection of chronic subclinical EP infections and for epidemiological surveys [36]. Several types of cELISA using purified recombinant antigens have been developed and are recommended by the United States Department of Agriculture (USDA) and World Organization for Animal Health (WOAH, formerly OIE) for international horse transport screening. Strongly positive serology is indicative of acute infection, but it must be noted that negative serology does not reliably exclude it. Also, antibody titers do not correlate well with the level of parasitemia [42]. Seroconversion occurs approximately 8–11 days after initial infection. Antibody titers tend to decrease after 2 to 3 months, though in the case of *T. equi*, they can remain stable for years [43,44].

EP represents an important equine health issue with significant economic impact. With no reliable chemoprophylaxis, prevention is limited to vector control with acaricides. It is a reportable disease according to WOAH guidelines, which recommend that screening for *B. caballi* and *T. equi* should be carried out according to the respective national guidelines before importation. This process has the potential for dramatic economic consequences for the international trade of equids [36,45]. The aim of this study was to assess the incidence of EP in Europe, utilizing samples submitted to a commercial laboratory, and to identify potential risk factors for pathogen contact and infection.

## 2. Materials and Methods

This retrospective study included both direct (PCR) and indirect detection methods (cELISA) for EP. All samples were sent to the commercial laboratory LABOKLIN GmbH & Co. KG (Bad Kissingen, Germany) by veterinarians in Europe between January 2008 and December 2021. Ethylenediaminetetra-acetic acid (EDTA) blood was analyzed using PCR for the small subunit ribosomal RNA gene ([46] (forward primer: 5′-CGGGATCCTTCACTCGCCGCTACT-3′; reverse primer: 5′-GCCTTGGGAAGTTTTGTGAACCTTA-3′). Following gel electrophoresis, PCR results were evaluated on a qualitative basis (negative/positive), with positive samples showing a PCR product at approximately 850 base pairs. Each PCR run included a negative and positive control. The confirmation of the positive results and species differentiation was conducted via Sanger sequencing and the comparison of the retrieved sequences via Basic Local Alignment Search Tool (BLAST).

For the serological testing of serum or plasma, the cELISA *Babesia caballi* Antibody Test Kit (VMRD, Inc., Pullmann, WA, USA) and the cELISA *Theileria equi* Antibody Test Kit (VMRD, Inc., Pullmann, WA, USA) were used according to manufacturer guidelines, which also specified a ≥40% cut-off for positivity. In all runs, positive and negative controls were included.

In the case of repeated samples from the same horse, only the results of either the first or the first positive sample were considered for statistical analysis. Where background data were available, an association analysis of EP test results and breed, sex, or age of the horses tested as well as the time of testing was performed. Seasonality was defined as spring (March–May), summer (June–August), autumn (September–November), and winter (December–February). For geographical analysis, countries were grouped as follows: Central (Austria, Germany, and Switzerland), Northern (Denmark, Estonia, Finland, Great Britain, Lativia, Lithuania, and Sweden), Western (Belgium, France, Luxembourg, and the Netherlands), Eastern (Czech Republic, Hungary, Poland, and Slovakia), and Southern (Bulgaria, Greece, Italy, Malta, Portugal, Romania, Slovenia, and Spain) European countries. For breed classification, Icelandic horses were grouped with ponies due to similarities in appearance/anatomy and husbandry.

According to the terms and conditions of the laboratory LABOKLIN, no special permission from animal owners or the animal welfare commission was needed as samples were submitted by veterinarians asking for PCR- and/or antibody-testing for EP. No samples were recruited exclusively for this study.

SPSS for Windows (Version 29.0; SPSS Inc., Armonk, NY, USA) was used for statistical analysis, with a significance threshold of *p* ≤ 0.05. The 95% confidence intervals (CI) for the percentages of horses with positive PCR and serological testing were calculated using the Wilson procedure, including correction for continuity. All metric parameters were checked for normality through Kolmogorov–Smirnov testing. Fisher’s exact tests were used to compare test results between the different groups of age (<9 years and ≥9 years, chosen by the median of the study population of 9 years), sex, breeds, seasonality, and regionality. Binary bivariate (also called “simple”) and multiple logistic regression analyses were performed to determine the effects of age, sex, breed, seasonality, regionality, and years of testing. For logistic regression analysis, PCR and serological results were included as the dichotomous dependent variables, while age group (<9 years vs. ≥9 years), sex (male vs. female), breed categories (one individual category vs. all other categories), season (one individual season vs. all other seasons), regionality (one individual region vs. all other regions), or year were entered as categorical variables. Odds ratios (OR) were calculated to identify potential risk factors for pathogen contact and/or infection.

For the subset of affected horses from Germany, a map was created to visualize their geographic origin (based on the postal code of the treating veterinarian) in relation to the occurrence of *Dermacentor reticulatus* and *Dermacentor marginatus* ticks. Both maps were created in R. v. 4.2.1 (R Core Team, 2022), with geographical data retrieved from the rworldmap package [47] and the Database of Global Administrative Areas (www.gadm.org, accessed on 10 March 2024). The records of *Dermacentor* occurrence were based on a citizen science study [48,49].

## 3. Results

### 3.1. Molecular and Serological Testing for EP

This study included samples from 4060 horses. PCR results were available for 1589/4060 horses (39.1%), 154 (9.7%) of which were positive. Serological testing was carried out for 2591/4060 horses (63.8%), with antibodies to *T. equi* and *B. caballi* detected in 393/2591 (15.2%) and 175/2578 (6.8%) horses, respectively. There were 46 cases of positive PCR results with species differentiation of which *T. equi* was identified in 32 (69.6%) and *B. caballi* in 14 horses (30.4%).

In 120/4060 horses (3.0%), PCR and serological testing was performed in parallel. Out of these 120 horses, 76 tested both PCR- and serologically negative (63.3%), 23 PCR-negative and serologically positive (19.2%), 18 both PCR- and serologically positive (15.0%), and 3 PCR-positive and serologically negative (2.5%).

### 3.2. Signalement

Information on the breed was available for 1806/4060 horses (44.5%) of 63 different breeds, mostly Pura Raza Espanol horses (*n* = 338), American Quarter horses (*n* = 90), Icelandic horses (*n* = 87), and Lusitano (*n* = 81). For statistical analysis, purebred horses were assigned to one of four groups: warmblood horses, heavy horses, ponies, and thoroughbreds (Table 1).

The age was known for 1856/4060 horses, with a median of 9.0 years (45.7%, minimum 4.0 months, maximum 36 years; standard deviation 6.2 years). For further analysis, the study population was divided at the median into two age groups (<9 years and ≥9 years) (Table 2).

The sex was known in 2573/4060 horses (63.4%). A total of 1062/2573 horses (41.3%) with known sex were tested by means of PCR, 1606/2573 (62.4%) by means of *T. equi* cELISA, and 1596/2573 (62.0%) by means of *B. caballi* cELISA (Table 2). Among male horses, 58/649 (8.9%) tested positive by means of PCR (intact: 23/207, 11.1%; geldings: 35/442, 7.9%; Fisher’s exact test *p* = 0.187), 70/1023 (6.8%) by means of *B. caballi* cELISA (intact: 32/442, 7.2%; geldings: 38/581, 6.5%; Fisher’s exact test *p* = 0.708), and 155/1029 (15.1%) by means of *T. equi* cELISA (intact: 83/445, 18.7%; geldings: 72/584, 12.3%; Fisher’s exact test *p* = 0.006).

The bivariate logistic regression analysis evaluating the statistically significant impact of age, sex, years of testing, categories of breeds, seasonality, and regionality on the results of PCR and serological testing is demonstrated in Table 3.

Male intact horses had higher odds than geldings of positive serology for *T. equi* (*B* = 0.489, *SE* = 0.175; Wald = 7.796; *p* = 0.005; OR = 1.630, 95% CI 1.157–2.298) but not *B. caballi* (*B* = 0.109, *SE* = 0.249; Wald = 0.192; *p* = 0.661; OR = 1.115, 95% CI 0.685–1.816). No effect of castration was seen for PCR results in male horses (*B* = 0.374, *SE* = 0.283; Wald = 1.750; *p* = 0.186; OR = 1.454, 95% CI 0.835–2.530).

### 3.3. Regionality, Seasonality, and Years of Testing

Data regarding the origin countries of the submitted samples and date of testing was available for all 4060 horses included in the study (Table 4 and Table S1).

The odds for a positive serology increased by 6.8% for *B. caballi* (*p* = 0.008) and by 9.5% for *T. equi* (*p* < 0.001) each year from 2008 to 2021. This trend was also seen for PCR testing (OR = 1.055); however, it was not statistically significant (*p* = 0.056) (Table 3). Dividing the timeframe of the study in three individual parts (2008–2012, 2013–2017, and 2018–2021), statistically significant impacts were demonstrated for PCR (*p* = 0.007) and serological testing (*p* < 0.001 each) (Table 5).

There was no statistically significant association between seasonality and the results of PCR testing (Table 3). A detailed breakdown of seasonality analysis by month for all three diagnostic assays can be found in Table S2.

In a multiple regression analysis, horses < 9 years had 77.4% higher odds of testing positive by means of PCR than those ≥9 years (*N* = 803, *p* = 0.016). In *B. caballi* cELISA testing, 47.8% higher odds were demonstrated for the timeframe of 2016 to 2022 compared to 2008 to 2015 (*N* = 1383, *p* = 0.009), and lower odds for spring and autumn compared to summer and winter (*N* = 1383, *p* = 0.007) (Table S3).

### 3.4. Geographic Origin of Affected Horses in Germany from 2008 to 2021

**Figure 1:**
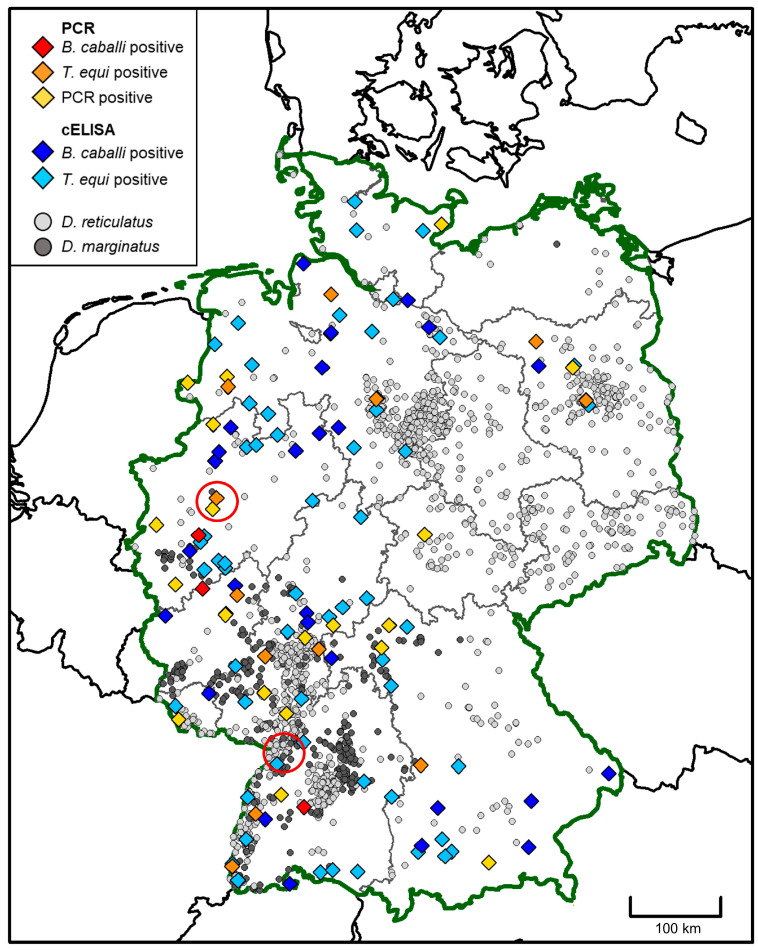
Geographic origin of horses tested positive for equine piroplasmosis by means of PCR or cELISA in Germany from 2008 to 2021, as inferred from the postal code of the submitting veterinarian. The occurrence of the vector ticks *Dermacentor reticulatus* and *Dermacentor marginatus* as determined by a citizen science study is shown as grey dots (data from Drehmann et al., 2020 [48] and Springer et al., 2022 [49]). Areas where presumably autochthonous EP cases were reported previously (Scheidemann et al., 2003 [34], Dirks et al., 2021 [33]) are encircled in red.

The geographic origin of samples tested positive by means of piroplasm-specific PCR within Germany is plotted together with data on the distribution of *Dermacentor* ticks in Figure 1.

## 4. Discussion

During the individual timeframes of the study (2008–2012, 2013–2017, and 2018–2021), a statistically significant increase in the detection frequency of EP by means of PCR and serology was apparent (Table 5). The odds for positive serological results for *B. caballi* and *T. equi* serology increased by 6.8% and 9.5% each year, respectively. EP infections can incur great costs for private and commercial owners [36,45], and their rising incidence signifies a great potential economic impact. There was a notable predominance of *T. equi* in the subset of samples with sequencing results, which may be due to the pathogen’s long patency [43,50].

Epidemiological studies in horses have consistently reported lower detection rates by means of molecular than serological methods [51,52,53,54]. Especially for *T. equi*, antibody titers can remain stable for years [43,44], and positive titers can only be interpreted as previous pathogen contact. However, our data showed comparable frequencies of positive results for PCR testing (9.7%) and serology (*T. equi*: 15.2%, *B. caballi*: 6.8%). This may be due to the nature of the samples sent in, as PCR testing is indicated for sick horses with clinical signs suspicious for EP while serological testing is most often used for screening and/or travelling. Moreover, PCR testing is more sensitive than any other diagnostic assay for chronic infections with EP. It is recommended to use PCR testing in addition to microscopy and serological assays [36]. Other potential influences on the percentages of horses tested PCR- and serologically positive may include changes in climate, resulting in the expansion of competent tick vectors into previously non-endemic areas, as demonstrated for *D. reticulatus* in Germany [49], the increased transport and travel of horses within Europe, and/or changes in husbandry associated with more opportunities for vector contact, like pasturing. Variation in climate has been documented to influence the seropositivity of EP [55], via both vector occurrence and activity [56]. This might be an underlying mechanism for the dynamic movement of detection rates seen over the years. Neither seasonality nor months of testing were associated with either molecular or serological test results for EP in this study. However, several tick species with different activity profiles can be competent vectors for EP [2]. The issue of endemisation of EP in Central Europe has been discussed in several studies. In Germany, Austria, and Switzerland, the monitoring of tick activity and strict measures to prevent the spread of equine piroplasms within local tick populations have been recommended [57].

### 4.1. Analysis of Risk Factors

#### 4.1.1. Regionality

Differences in EP prevalence within Europe are predominantly due to the presence or abundance of competent tick vectors, in addition to host activities, the living conditions of the horses, management practices, and regional vector control programs [36]. Southern Europe is a highly endemic area for EP; consequently, samples from horses in Southern Europe had 45% higher odds of positive PCR testing as well as 152% higher odds of positive *T. equi* serology. In general, *T. equi* is more prevalent than *B. caballi*, which this study could also confirm. Horses infected with *T. equi* are considered life-long carriers, while *B. caballi* is usually cleared from the blood circulation within four years of resolution [15].

#### 4.1.2. Age

A previous study reported an association between EP prevalence and the increasing age of horses [36]. Our data showed the opposite: younger horses (<9 years) had doubled odds of EP detection by means of PCR than older horses (≥9 years); however, this might be a spurious result and needs to be reevaluated in further studies. Several experimental and epidemiological studies have reported a correlation between age and *T. equi* infections [29,50], and *T. equi* infections tend to persist while *B. caballi* infections are eliminated with increasing age [50,55]. However, one study showed no influence of age on EP prevalence [55]. Our data showed a trend of higher seroprevalence rates for *T. equi* (OR = 1.333) and *B. caballi* (OR = 1.156) in older horses, though this was not statistically significant. EP seroprevalence may rise with increasing age due to a higher cumulative risk of pathogen contact and long lasting seropositivity (at least for *T. equi*), with the above-mentioned limitations.

#### 4.1.3. Sex

A sex-dependent difference in susceptibility to protozoan infections has been reported in horses, possibly due to different levels of sex hormones [36,58]. However, different studies show contradictory effects of sex on infection rates [36]. Our data did not show a statistically significant association of sex and PCR or serology results. Regarding castration status in male horses, male intact horses had higher odds than geldings of positive serology for *T. equi* in our study (*p* = 0.005; OR = 1.630). Our findings are consistent with a previous study showing not only approximately three times higher odds of *T. equi* infection but also higher tick burden in intact male horses than in geldings, which was explained by a potential influence of sex-specific husbandry techniques [50]. Forty-six percent higher odds of testing positive by means of *Babesia* spp. PCR were demonstrated for male compared to female dogs [59]. These differences may be linked to physiological effects of sex hormones, in particular testosterone, or to behavioral effects, e.g., the higher exposure of one sex due to a different habitat preference. Castration reverses the immunosuppressive effects of testosterone [60]. In conclusion, it remains unclear why male intact horses had a higher predisposition for *T. equi* infections, and further studies are needed to elucidate this.

#### 4.1.4. Breed

This is the first study to compare the impact of breed categories on EP incidence. Significantly different odds were demonstrated for heavy horses in *B. caballi* serology (*p* = 0.016, OR = 2.239) and for warmbloods (*p* = 0.025, OR = 1.602) as well as ponies (*p* = 0.007, OR = 0.340) in *T. equi* serology compared to all other categories, respectively. As thoroughbreds are most often used in sports linked to national and international travel, it was surprising that no statistically significant impact on molecular and serological results could be demonstrated, especially in comparison to ponies and heavy horses. On the other hand, horses used in sports are most often kept in stables and therefore are not at high risk of tick attachment.

On the other hand, heavy horses had higher odds of a positive *B. caballi* cELISA. However, our findings are difficult to generalize due to the small number of heavy horses included in the study and the lack of further background history regarding husbandry or import. Overall, risk factors for the different susceptibility of individual breeds have not been investigated but may include hair coats and colors as well as living conditions.

### 4.2. Endemisation in Germany

There is a growing risk of EP endemisation in previously non-endemic countries like Germany, particularly as the EP vector *D. reticulatus* has recently undergone a dramatic range expansion [48,49,61,62]. For example, *B. caballi* has been recently detected in two *D. reticulatus* ticks in Belgium [63]. The endemisation risk is likely even greater for *B. caballi* than for *T. equi* due to the potential for transovarial transmission in the tick vector [64]. Plotting the geographic origin of horses diagnosed with EP in this study according to the postal code of the treating veterinarian together with *Dermacentor* tick habitats highlights areas particularly at risk of endemisation in this country. An overlap of horses with positive PCR results and the occurrence of *Dermacentor* ticks was particularly noted in southwestern Germany, where both *D. reticulatus* and *D. marginatus* occur [48,49]. Two of the three presumably autochthonous EP cases reported in Germany to date occurred near the city of Karlsruhe in southwestern Germany [33]. The last was reported near Dortmund in western Germany [34], which was also the origin of several horses with positive PCR results in this study. Still, it should be noted that comparatively few samples were submitted from eastern Germany, where especially *D. reticulatus* is widely distributed [48,49]. The endemisation risk for the eastern part of Germany may therefore be underestimated by our data. In dogs, the prediction mapping of *Babesia* spp.-positive PCR results from 2007 to 2020 on a five-digit postal code level revealed a pathogen predominance in northwestern, eastern, and southern parts of Germany [59]. *Babesia canis* is transmitted to dogs by *D. reticulatus* ticks, and canine infections are closely related to vector habitats [59,65,66]. Consequently, a higher risk of canine infections in southwestern, northeastern, and western parts of Germany might be linked to the vector distribution as well as the import of infected dogs. Of note, the above-mentioned range expansion of *D. reticulatus* includes for the first-time northwestern Germany [48,49]. The high numbers of horses with EP in this area are of concern regarding potential EP endemisation.

### 4.3. Diagnostic Assays

Several serological assays have been developed to diagnose chronic EP infections, including the complement fixation test, enzyme-linked immunosorbent assays, immunochromatographic tests, Western blots, and indirect immunofluorescence assays [8,67]. While highly positive titers indicate chronic EP, negative results cannot exclude an infection. This suggests that our data may even underestimate the number of affected horses. No direct relationship has been demonstrated between antibody titers and the level of parasitemia [42]. For the detection of *T. equi*, cELISA is reportedly the most sensitive serological assay [68]. This cELISA is approved by the WOAH as one of the regulatory tests for screening horses for both parasites, *B. caballi and T.equi*, prior to international transport to countries considered non-endemic [14].

### 4.4. Limitations

The limitations of this study are mainly linked to the retrospective study design with missing data regarding background history, ectoparasite prophylaxis, travel, and the reason for molecular and serological testing. The study population was preselected due to the fact that samples were submitted by veterinarians for screening or due to the clinical suspicion of EP, which could not be further investigated due to missing anamnesis. Therefore, the data of this study should not be misinterpreted as prevalence data for EP in horses in Europe. Mapping was based on the postal codes of the veterinarians requesting the tests, assuming that these reflect the areas where the horses were kept, as more precise data on the horses’ origin were not available. While this may have introduced some uncertainty into the map, the regional analysis was most likely not affected as the data were clustered based on countries in Europe and therefore at a larger spatial scale than the postal codes. Nevertheless, all the limitations mentioned may have impacted the reported percentages of affected horses.

## 5. Conclusions

The long-term approach from 2008 to 2021, including a high number of horses tested by means of PCR and/or serologically, in combination with the analysis of risk factors for infection and/or pathogen contact were the major novelties of our study, with 11.6% of the horses tested PCR-positive and 14.7% serologically positive. The frequency of disease was additionally associated with habitats of the vectors explaining the statistically significant positive association of regionality in PCR testing with Southern Europe. In addition to travelling and import, changes in climatic conditions, especially in Central, Northern, and Western Europe, might contribute to the spread and rising importance of equine piroplasmosis in Europe, which is additionally underlined by the results of our study and may have significant economic impact. The results of our study may also lead to further investigation regarding the effects of sex as well as castration status and breeds. Especially the investigation of individual breeds, which was included for the first time in such a study design on equine piroplasmosis, may provide interesting results which should be further investigated.

Significant effects of regionality were seen in the results of PCR testing. This finding was significant for *T. equi* but not so for *B. caballi* seropositivity. The consistent increase in *T. equi* detected by means of PCR and cELISA over the study timeframe may indicate the rising importance of EP in Europe.

In conclusion, EP should be considered as a potential differential diagnosis in endemic countries and in horses imported from endemic countries. Due to the expansion of potential vectors like *Dermacentor* ticks, the endemic areas in Europe may have to be redefined in the future, and autochthonous infections in Central European countries like Germany should not be excluded.

## Figures and Tables

**Table 1:** Breed-dependent test results in 1806 pure-bred horses in Europe tested by means of piroplasm specific PCR, *Babesia* (*B*.) *caballi* cELISA, and/or *Theileria* (*T*.) *equi* cELISA (*n* tested positive/*N* total (%) [95% CI lower limit; 95% CI upper limit]).

Categories	*n*/*N* Total (%)	Piroplasm Specific PCR	*B. caballi* cELISA	*T. equi* cELISA
Warmblood horses	1299/1806 (71.9)	43/477 (9.0 [6.8; 11.9])	49/872 (5.6 [4.3; 7.4])	146/878 (16.6 [14.3; 19.2])
Heavy horses	185/1806 (10.2)	6/93 (6.5 [2.9; 13.4])	12/99 (12.1 [7.1; 20.0])	12/100 (12.0 [7.0; 19.8])
Ponies	185/1806 (10.2)	5/75 (6.7 [2.9; 14.7])	9/112 (8.0 [4.3; 14.6])	7/113 (6.2 [3.0; 12.2])
Thoroughbreds	137/1806 (7.6)	11/73 (15.1 [8.6; 25.0])	3/67 (4.5 [1.5; 12.4])	12/67 (17.9 [10.6; 28.7])
Total	1806/1806 (100)	65/718 (9.1 [7.2; 11.4])	73/1150 (6.3 [5.1; 7.9])	177/1158 (15.3 [13.3; 17.5])
Fisher’s exact test ^1^	-	*p* = 0.375	*p* = 0.079	*p* = 0.013 *

cELISA: competitive enzyme-linked immunosorbent assay; CI: confidence interval; PCR: polymerase chain reaction. ^1^ Fisher’s exact test; * *p* < 0.05.

**Table 2:** Known age and sex in horses tested by means of piroplasm-specific PCR, *Babesia caballi* cELISA, and/or *Theileria equi* cELISA (*n* tested positive/*N* total (%) [95% CI lower limit; 95% CI upper limit]).

	Age (*N* = 1856)		*p* ^1^
	<9 Years	≥9 Years	
Piroplasm-specific PCR	42/348 (12.1 [9.1; 15.9])	24/385 (6.2 [4.2; 9.1])	0.007 *
*Theileria equi* cELISA	107/716 (14.9 [12.5; 17.7)	56/481 (11.6 [9.1; 14.8])	0.122
*Babesia caballi* cELISA	44/707 (6.2 [4.7; 8.3])	26/479 (5.4 [3.7; 7.8])	0.617
	**Sex (*N* = 2573)**		***p* ^1^**
	**Female**	**Male**	
Piroplasm-specific PCR	38/413 (9.2 [6.8; 12.4])	58/649 (8.9 [7.0; 11.4])	0.913
*Theileria equi* cELISA	79/577 (13.7 [11.1; 16.7])	155/1029 (15.1 [13.0; 17.4])	0.507
*Babesia caballi* cELISA	35/573 (6.1 [4.4; 8.4])	70/1023 (6.8 [5.5; 8.6])	0.600

cELISA: competitive enzyme-linked immunosorbent assay; CI: confidence interval; PCR: polymerase chain reaction. ^1^ Fisher’s exact test; * *p* < 0.05.

**Table 3:** Bivariate logistic regression analyses in horses tested by means of piroplasm-specific PCR, *Babesia caballi* cELISA, and/or *Theileria equi* cELISA.

	*N*	*B*	*SE*	Wald	*p*	Odds Ratio	95%-CI for Odds Ratio	
							Lower Limit	Upper Limit
Piroplasm-specific PCR								
Age (<9 years) *	733	0.725	0.267	7.348	0.007 *	2.065	1.222	3.487
Sex (male)	1062	−0.032	0.219	0.021	0.884	0.968	0.631	1.487
Year (year)	1589	0.053	0.028	3.650	0.056	1.055	0.999	1.114
Breed (Warmblood)	718	−0.014	0.275	0.003	0.960	0.986	0.575	1.690
Breed (Heavy horses)	718	−0.413	0.444	0.867	0.352	0.662	0.277	1.579
Breed (Ponies)	718	−0.365	0.482	0.573	0.449	0.694	0.270	1.786
Breed (Thoroughbreds)	718	0.664	0.357	3.461	0.063	1.942	0.965	3.907
Season (Spring)	1589	0.029	0.201	0.021	0.884	1.030	0.695	1.525
Season (Summer)	1589	0.225	0.176	1.642	0.200	1.252	0.888	1.767
Season (Autumn)	1589	−0.079	0.198	0.161	0.689	0.924	0.626	1.362
Season (Winter)	1589	−0.296	0.236	1.573	0.210	0.744	0.469	1.181
Region (North)	1589	−0.280	0.473	0.350	0.554	0.756	0.299	1.911
Region (South) *	1589	0.372	0.170	4.769	0.029 *	1.451	1.039	2.026
Region (Central) *	1589	−0.483	0.179	7.265	0.007 *	0.617	0.434	0.876
Region (West)	1589	−0.923	0.727	1.610	0.204	0.397	0.096	1.653
Region (East) *	1589	0.473	0.239	3.939	0.047 *	1.605	1.006	2.562
*Babesia caballi* cELISA								
Age (<9 years)	1186	0.145	0.255	0.325	0.569	1.156	0.702	1.905
Sex (male)	1596	0.121	0.241	0.322	0.570	1.129	0.742	1.717
Year (years) *	2578	0.066	0.025	7.071	0.008 *	1.068	1.017	1.121
Breed (Warmblood)	1150	−0.462	0.259	3.173	0.075	0.630	0.379	1.047
Breed (Heavy horses) *	1150	0.806	0.335	5.786	0.016 *	2.239	1.161	4.316
Breed (Ponies)	1150	0.285	0.371	0.591	0.442	1.330	0.643	2.750
Breed (Thoroughbreds)	1150	−0.388	0.604	0.414	0.520	0.678	0.208	2.214
Season (Spring)	2578	0.251	0.187	1.800	0.180	1.286	0.891	1.857
Season (Summer)	2578	−0.350	0.187	3.497	0.061	0.705	0.488	1.017
Season (Autumn)	2578	0.278	0.161	2.968	0.085	1.320	0.963	1.810
Season (Winter)	2578	−0.263	0.212	1.527	0.217	0.769	0.507	1.166
Region (North)	2578	−0.537	0.350	2.355	0.125	0.584	0.294	1.160
Region (South)	2578	0.313	0.160	3.841	0.050	1.367	1.000	1.869
Region (Central)	2578	−0.222	0.162	1.876	0.171	0.801	0.583	1.101
Region (West)	2578	0.109	0.259	0.178	0.673	1.115	0.672	1.851
Region (East)	2578	0.059	0.310	0.036	0.850	1.060	0.577	1.948
*Theileria equi* cELISA								
Age (<9 years)	1197	0.288	0.177	2.654	0.103	1.333	0.943	1.885
Sex (male)	1606	0.111	0.149	0.558	0.455	1.118	0.834	1.498
Year (years) *	2591	0.091	0.018	26.684	<0.001 *	1.095	1.058	1.134
Breed (Warmblood) *	1158	0.471	0.211	4.993	0.025 *	1.602	1.060	2.422
Breed (Heavy horses)	1158	−0.304	0.319	0.906	0.341	0.738	0.395	1.380
Breed (Ponies) *	1158	−1.079	0.399	7.309	0.007 *	0.340	0.155	0.743
Breed (Thoroughbreds)	1158	0.203	0.330	0.377	0.539	1.224	0.642	2.336
Season (Spring)	2591	0.015	0.139	0.011	0.915	1.015	0.773	1.332
Season (Summer)	2591	−0.139	0.124	1.254	0.263	0.870	0.683	1.110
Season (Autumn)	2591	−0.024	0.117	0.043	0.836	0.976	0.776	1.228
Season (Winter)	2591	0.188	0.133	2.001	0.157	1.207	0.930	1.567
Region (North) *	2591	−0.773	0.259	8.898	0.003 *	0.462	0.278	0.767
Region (South) *	2591	0.925	0.111	69.229	<0.001 *	2.521	2.027	3.134
Region (Central)	2591	−0.622	0.119	27.449	<0.001 *	0.537	0.426	0.678
Region (West)	2591	−0.351	0.209	2.828	0.093	0.704	0.468	1.060
Region (East)	2591	−0.082	0.228	0.131	0.717	0.921	0.590	1.438

B: unstandardized regression weight; cELISA: competitive enzyme-linked immunosorbent assay; CI: confidence interval; SE: standard deviation to the mean; PCR: polymerase chain reaction. Degrees of freedom were 1 for all Wald statistics. * *p* < 0.05.

**Table 4:** Regional distribution in 4060 horses in Europe tested by means of piroplasm-specific PCR, *Babesia* (*B*.) *caballi* cELISA, and/or *Theileria* (*T*.) *equi* cELISA (*n* tested positive/*N* total (%) [95% CI lower limit; 95% CI upper limit]).

Region	*n*/*N* Total (%)	PCR	*B. caballi* cELISA	*T. equi* cELISA
Central Europe	1695/4060 (41.7)	51/690 (7.4 [5.7; 9.6])	64/1070 (6.0 [4.7; 7.6])	116/1079 (10.8 [9.0;12.7])
Eastern Europe	336/4060 (8.3)	24/172 (14.0 [9.6; 19.9])	12/168 (7.1 [4.1; 12.1])	24/169 (14.2 [9.7; 20.3])
Western Europe	289/4060 (7.1)	2/48 (4.2 [1.2; 14.0])	18/242 (7.4 [4.8; 11.4])	28/244 (11.5 [8.1; 16.1])
Northern Europe	267/4060 (6.6)	5/66 (7.6 [3.3; 16.5])	9/213 (4.2 [2.0; 7.9])	17/213 (8.0 [5.0; 12.4])
Southern Europe	1473/4060 (36.3)	72/613 (11.7 [9.4; 14.5])	72/885 (8.1 [6.5; 10.1])	208/886 (23.5 [20.8; 26.4])
Total	4060/4060 (100)	154/1589 (9.7 [8.3; 11.2])	175/2578 (6.8 [5.9; 7.8])	393/2591 (15.2 [13.8; 16.6])
Fisher’s exact test ^1^	-	*p* = 0.014 *	*p* = 0.193	*p* < 0.001 *

cELISA: competitive enzyme-linked immunosorbent assay; CI: confidence interval; PCR: polymerase chain reaction. Central Europe: Austria, Germany, and Switzerland. Eastern Europe: Czech Republic, Hungary, Poland, and Slovakia. Western Europe: Belgium, France, Luxembourg, and the Netherlands. Northern Europe: Denmark, Estonia, Finland, Great Britain, Lativia, Lithuania, and Sweden. Southern Europe: Bulgaria, Greece, Italy, Malta, Portugal, Romania, Slovenia, and Spain. ^1^ Fisher’s exact test; * *p* < 0.05.

**Table 5:** Percentages of horses tested positive by means of piroplasm-specific PCR, *Babesia caballi* cELISA, and/or *Theileria equi* cELISA from 2008 to 2021, sorted by three different timeframes (*n* tested positive/*N* total (%, [95% confidence interval lower limit; 95% confidence interval upper limit]).

Timeframes	PCR	*B. caballi* cELISA	*T. equi* cELISA
2008–2012	4/141 (2.8 [1.1; 7.1])	17/302 (5.6 [3.5; 8.8])	28/308 (9.1 [6.4; 12.8])
2013–2017	56/533 (10.5 [8.2; 13.4])	36/818 (4.4 [3.2; 6.0])	85/825 (10.3 [8.4; 12.6])
2018–2021	94/915 (10.3 [8.5; 12.4])	122/1458 (8.4 [7.1; 9,9])	280/1458 (19.2 [17.3; 21.3])
Total	154/1589 (9.7 [8.3; 11.2])	175/2578 (6.8 [5.9; 7.8])	393/2591 (15.2 [13.8; 16.6])
Fisher’s exact test ^1^	*p* = 0.007 *	*p* < 0.001 *	*p* < 0.001 *

cELISA: competitive enzyme-linked immunosorbent assay; CI: confidence interval; PCR: polymerase chain reaction. ^1^ Fisher’s exact test; * *p* < 0.05.

## Data Availability

Data are contained within the article and Supplementary Materials.

## Supplementary Materials

The following supporting information can be downloaded at: https://www.mdpi.com/article/10.3390/microorganisms12040816/s1, Table S1: Number of horses and percentages of horses tested positive for equine piroplasmosis by molecular and serological testing in the laboratory LABOKLIN from 2008 to 2021 (*n* tested positive/*N* total (%, [95% confidence interval lower limit; 95% confidence interval upper limit]); Table S2: Number of horses and percentages of horses tested positive for equine piroplasmosis by molecular and serological testing in the laboratory LABOKLIN from 2008 to 2021 sorted by months of testing (*n* tested positive/*N* total (%, [95% confidence interval lower limit; 95% confidence interval upper limit]); Table S3: Multiple logistic regression analysis in 658 horses tested by piroplasm-specific PCR, in 1038 horses tested by *Babesia caballi* cELISA, and in 1048 horses tested by *Theileria equi* cELISA from 2008 to 2021 in the laboratory LABOKLIN.
