# Supplementary material for: Molecular and Serological Detection of Vector-Borne Pathogens Responsible for Equine Piroplasmosis in Europe between 2008 and 2021
Source: Microorganisms. 2024 Apr 17;12(4):816. doi: 10.3390/microorganisms12040816 (PMC11051957; doi:10.3390/microorganisms12040816)
Supplement: Supplementary file 1 [file microorganisms-12-00816-s001.zip › microorganisms-2964695-supplementary.pdf]

Table S1. Number of horses and percentages of horses tested positive for equine piroplasmosis by molecular and serological testing in the laboratory LABOKLIN from 2008 to 2021 (*n* tested positive / *N* total (% [95% CI lower limit;95% CI upper limit])

Year	Piroplasm-specific PCR	*Babesia caballi* cELISA	*Theileria equi* cELISA
2008	2/35 (5.7 [1.6;18.6])	12/34 (35.3 [21.5;52.1])	7/34 (20.6 [10.4;36.8])
2009	1/25 (4.0 [0.7;19.5])	2/46 (4.3 [1.2;14.5])	8/46 (17.4 [9.1;30.7])
2010	0/26 (0 [0;12.9])	2/59 (3.4 [0.9;11.5])	3/64 (4.7 [1.6:12.9])
2011	0/20 (0 [0;16.1])	0/69 (0 [0;5.2])	6/69 (8.7 [4.0;17.7])
2012	1/35 (2.9 [0.5;14.5])	1/94 (1.1 [0.2;5.8])	4/95 (4.2 [1.6;10.3])
2013	3/62 (4.8 [1.7;13.3])	5/147 (3.4 [1.5;7.7])	18/147 (12.2 [7.9;18.5])
2014	6/55 (10.9 [5.1;21.8])	8/104 (7.7 [3.9;14.5])	11/110 (10.0 [5.7;17.0])
2015	21/151 (13.9 [9.3;20.3])	2/176 (1.1 [0.3;4.0])	9/176 (5.1 [2.7;9.4])
2016	16/110 (14.5 [9.2;22.3])	7/173 (4.0 [2.0;8.1])	16/173 (9.2 [5.7;14.5])
2017	10/155 (6.5 [3.5;11.5])	14/218 (6.4 [3.9;10.5])	31/219 (14.2 [10.2;19.4])
2018	16/171 (9.4 [5.8;14.7])	12/255 (4.7 [2.7;8.0])	50/255 (19.6 [15.2;24.9])
2019	20/201 (10.0 [6.5;14.9])	31/303 (10.2 [7.3;14.2])	60/303 (19.8 [15.7;24.7])
2020	28/240 (11.7 [8.2;16.3])	22/307 (7.2 [4.8;10.6])	69/307 (22.5 [18.2;27.5])
2021	30/303 (9.9 [7.0;13.8])	57/593 (9.6 [7.5;12.3])	101/593 (17.0 [14.2;20.3])
**Total**	**154/1589 (9.7 [8.3;11.3])**	**175/2578 (6.8 [5.9;7.8])**	**393/2591 (15.2 [13.8;16.6])**
**Fisher's exact test**	$P = 0.119$	$P < 0.001$	$P < 0.001$

cELISA: competitive enzyme-linked immunosorbent assay; CI: confidence interval; PCR: polymerase chain reaction

Table S2. Number of horses and percentages of horses tested positive for equine piroplasmosis by molecular and serological testing in the laboratory LABOKLIN from 2008 to 2021 sorted by months of testing (*n* tested positive / *N* total (% [95% CI lower limit;95% CI upper limit])

Month	Piroplasm-specific PCR	*Babesia caballi* cELISA	*Theileria equi* cELISA
January	8/98 (8.2 [3.6;15.5])	5/119 (4.2 [0.8;5.8])	15/119 (12.6 [7.2;19.9])
February	8/112 (7.1 [3.1;13.6])	15/153 (9.8 [5.6;15.7])	28/153 (18.3 [12.5;25.4])
March	16/122 (13.1 [7.7;20.4])	13/154 (8.4 [4.6;14.0])	26/154 (16.9 [11.3;23.8])
April	8/111 (7.2 [3.2;13.7])	14/138 (10.1 [5.7;16.4])	19/139 (13.7 [8.4;20.5])
May	12/131 (10.7 [4.8;15.5])	13/198 (6.6 [3.5;11.0])	31/203 (15.3 [10.6;21.0])
June	23/187 (12.3 [8.0;17.9])	13/165 (8.4 [4.3;13.1])	26/165 (15.8 [10.6:22.2])
July	27/211 (12.8 [8.6;18.1])	10/227 (4.4 [2.1;8.0])	39/233 (16.7 [12.2;22.2])
August	8/127 (6.3 [2.8;12.0])	16/342 (4.7 [2.7;7.5])	38/342 (11.1 [8.0;14.9])
September	9/130 (6.9 [3.2;12.7])	24/268 (9.0 [5.8;13.0])	52/268 (19.4 [14.8;24.7])
October	14/142 (9.9 [5.5;16.0))	22/275 (8.0 [5.1;11.9])	38/275 (13.8 [10.0;18.5])
November	14/131 (10.7 [6.0;17.3])	22/306 (7.2 [4.6;10.7])	37/306 (12.1 [8.7;16.3])
December	7/87 (8.0 [3.3;15.9])	8/233 (3.4 [1.5;6.7])	44/234 (18.8 [14.0;24.4])
**Total**	**154/1589 (9.7 [8.3;11.3])**	**175/2578 (6.8 [5.9;7.8])**	**393/2591 (15.2 [13.8;16.6])**
**Chi-squared test**	$P = 0.481$	$P = 0.077$	$P = 0.141$

cELISA: competitive enzyme-linked immunosorbent assay; CI: confidence interval; PCR: polymerase chain reaction

Table S3. Multiple logistic regression analysis in 658 horses tested by piroplasm-specific PCR, in 1038 horses tested by *Babesia caballi* cELISA, and 1048 horses tested by *Theileria equi* cELISA from 2008 to 2021 in the laboratory LABOKLIN

	B	***SE***	**Wald**	***P***	**Odds Ratio**	**95%-CI for Odds Ratio**	
						**Lower bound**	**Upper Bound**
**Piroplasm-specific PCR (*n* = 658)**							
Sex (male)	0.372	0.311	1.431	0.232	1.451	0.789	2.669
**Age (< 9 years)**	**0.704**	**0.292**	**5.814**	**0.016**	**2.022**	**1.141**	**3.584**
Years (years)	-0.052	0.076	0.476	0.490	0.949	0.818	1.101
Timeframe (2016-2022)	0.810	0.607	1.778	0.182	2.247	0.683	7.391
Season (Spring/autumn)	0.247	0.289	0.727	0.394	1.280	0.726	2.256
***Babesia caballi* cELISA (*n* = 1038)**							
Sex (male)	0.380	0.290	1.717	0.190	1.462	0.828	2.580
Age (< 9 years)	0.102	0.271	0.140	0.708	1.107	0.651	1.882
Years (years)	0.044	0.075	0.334	0.564	1.045	0.901	1.211
Timeframe (2016-2022)	0.849	0.623	1.856	0.173	2.338	0.689	7.936
Season (Spring/autumn)	-0.517	0.273	3.593	0.058	0.596	0.349	1.018
***Theileria equi* cELISA (*n* = 1048)**							
Sex (male)	0.299	0.197	2.309	0.129	1.348	0.917	1.982
Age (< 9 years)	0.321	0.190	2.834	0.092	1.378	0.949	2.001
**Years (years)**	**0.146**	**0.054**	**7.327**	**0.007**	**1.157**	**1.041**	**1.285**
Timeframe (2016-2022)	0.129	0.419	0.094	0.759	1.137	0.500	2.585
Season (Spring/autumn)	0.073	0.183	0.161	0.688	1.076	0.752	1.539

B: unstandardized regression weight; cELISA: competitive enzyme-linked immunosorbent assay; CI: confidence interval; SE: standard deviation to the mean; PCR: polymerase chain reaction

Degrees of freedom were 1 for all Wald statistics
